# The PhyloPythiaS Web Server for Taxonomic Assignment of Metagenome Sequences

**DOI:** 10.1371/journal.pone.0038581

**Published:** 2012-06-20

**Authors:** Kaustubh Raosaheb Patil, Linus Roune, Alice Carolyn McHardy

**Affiliations:** 1 Max-Planck Research Group for Computational Genomics and Epidemiology, Max-Planck Institute for Informatics, Saarbrücken, Germany; 2 Department of Algorithmic Bioinformatics, Heinrich-Heine University, Düsseldorf, Germany; Baylor College of Medicine, United States of America

## Abstract

Metagenome sequencing is becoming common and there is an increasing need for easily accessible tools for data analysis. An essential step is the taxonomic classification of sequence fragments. We describe a web server for the taxonomic assignment of metagenome sequences with PhyloPythiaS. PhyloPythiaS is a fast and accurate sequence composition-based classifier that utilizes the hierarchical relationships between clades. Taxonomic assignments with the web server can be made with a generic model, or with sample-specific models that users can specify and create. Several interactive visualization modes and multiple download formats allow quick and convenient analysis and downstream processing of taxonomic assignments. Here, we demonstrate usage of our web server by taxonomic assignment of metagenome samples from an acidophilic biofilm community of an acid mine and of a microbial community from cow rumen.

## Introduction

A metagenome sequence sample is obtained by sequencing the DNA of a mixture of microorganisms from an environment of interest [1]. Identification of the taxonomic affiliation of DNA sequences, either for individual reads or assembled contigs, is an essential step prior to further analysis, such as characterization of the functional and metabolic capabilities of the sequenced microbial community [2]. Various taxonomic assignment methods exist, which can be divided into three categories: sequence composition-based, sequence alignment-based and hybrids; see [3], [4] and [5] respectively for examples. Sequence composition based methods use short substrings (k-mers) to represent a sequence as a vector of fixed length, which is used to assess similarity among sequences. Such a representation is known as a “genomic signature” and is more conserved between evolutionarily close species than distant species [6], [7]. Sequence alignment and phylogeny-based methods use sequence similarity as a measure of evolutionary relatedness between sequences. This approach is computationally more expensive compared to sequence composition, and thus requires more hardware resources for analysis of large datasets. Hybrid methods combine information from both sequence composition and alignment to assess similarity between sequences. From another perspective, taxonomic assignment methods can be categorized as either unsupervised or supervised methods. Unsupervised methods cluster the sequences based on a similarity measure and then assign a taxonomic affiliation to the clusters. Supervised methods, on the other hand, infer a taxonomic model using sequences of known taxonomic origin, which are then used for taxonomic assignment of novel metagenome sequences. Given that sufficient reference data for modeling are available, supervised methods are likely to be more accurate in taxonomic assignment than clustering techniques, as the effect of non-taxonomic signals, such as guanine and cytosine strand biases, on taxonomic assignment is minimized during model induction.

Recently we developed a new method PhyloPythiaS, which is a successor to the previously published software PhyloPythia [8], [9]. PhyloPythiaS exhibits high prediction accuracy and allows a rapid analysis of datasets with several hundred mega-bases or giga-bases. PhyloPythiaS was benchmarked on simulated and real data sets and shows good predictive performance. PhyloPythiaS shows notably reduced execution times in comparison to MEGAN [4] and PhymmBL [5] (85-fold and 106-fold respectively on a 13 Mb assembled metagenome sample), as no similarity searches are performed against large databases. It also shows better predictive performance on both simulated and real metagenome samples, in particular when limited amount of reference sequences from particular species are available (approximately 100 kb). While for short fragments, all methods perform less favorably than for fragments of 1 kb in length or more [2], similarity-based assignment with MEGAN has the lowest error rate for short fragments. PhyloPythiaS is freely available for non-commercial users and can be installed on a Linux-based machine [8].

PhyloPythiaS can be used in two different modes – generic and sample-specific. The generic model is suitable for the analysis of a metagenome sample, if no further information on the sample's taxonomic composition or relevant reference data are available. Assignment accuracy can be improved by creation and use of a sample-specific model, which includes clades for the abundant sample population that are inferred from the appropriate reference sequences. A sample-specific model is inferred from public sequence data combined with sequences with known taxonomic affiliation identified from the metagenome sample, along with a customized taxonomy. If a better match to the taxa in the metagenome sample is achieved, sample-specific models exhibit higher predictive accuracy, and have improved resolution to low-ranking clades and higher coverage in terms of assigned sequences compared to the generic model. Accurate assignments can be obtained based on ∼100 kb of reference sequence for a modeled sample population [8].

Here we present a web server for taxonomic sequence assignment for web-based use of PhyloPythiaS. The underlying functionality of the software is as we have described it before. For researchers with limited computational resources or who are not familiar with command line usage under Unix/Linux, web servers provide computational resources and a graphical user interface for convenient use. Furthermore, they allow a visual presentation of results for a quick overview and exploration of data sets. Several web servers for taxonomic assignment are available, such as the MG-RAST [10], WebCARMA [11] and the Naïve Bayes Classification (NBC) [12] web servers. Our server is unique in that it provides the ability to construct and use sample-specific models, besides enabling assignment with generic models. We illustrate taxonomic metagenome assignment with the generic and sample-specific modes of the web server by analyzing metagenome samples of an acidophilic biofilm community from acid mine drainage (AMD) [13] and of a cow rumen microbial community [14].

## Results

We demonstrate the functionality of the web server based on a taxonomic assignment of two metagenome sequence samples. For performance analysis, we assessed the consistency and taxonomic distance of assignments, as defined in [8]. A prediction for a sequence fragment was considered to be consistent if the fragment was either assigned to the correct clade or to a parental clade of the correct taxonomic label. The consistency was measured as the percentage of sequence fragments or base-pairs assigned correctly. Higher consistency in terms of assigned base-pairs than number of sequence fragments indicates that longer sequences are classified more consistently than short ones. As the consistency considers also assignments to parental clades to be correct, it is a ‘coarse’ performance measure. As a more ‘fine grained’ performance measure, we also calculated the taxonomic distance, based on the geodesic distance between the correct and predicted nodes in the reference taxonomy. Taken together, these two measures provide good qualitative assessment. A well performing method will produce assignments with both high consistency and low taxonomic distance. For clades of the analyzed samples, the average values of consistencies and taxonomic distances over the corresponding sequences are reported, in addition to average values for the entire sample. We also calculated accuracy values for clades at the genus-level and for higher taxonomic ranks, both in terms of the fraction of sequences and based on the fraction of base-pairs correctly assigned.

### Analysis of the Acid Mine Drainage (AMD) Data-set

We used our web server for the taxonomic assignment of a well-studied metagenome of an acidophilic biofilm community, sequenced with Sanger sequencing technology. The AMD community comprises five abundant species: Ferroplasma Types I and II, a Thermoplasmatales species (all Euryarchaeota), and Leptospirillum sp. Group I and II of the phylum Nitrospirae. The *test scaffolds* for the AMD metagenome were downloaded from the IMG/M portal (http://img.jgi.doe.gov/, taxon object ID 2001200000). These data comprise 1183 scaffolds and ∼10.83 Mb of DNA sequence. Draft genome assemblies, comprising 908 scaffolds overall, were created using sequencing coverage and nucleotide composition for the five populations of the AMD sample; the genome assemblies were then deposited at NCBI (accession numbers CH003520–CH004435). We mapped the AMD scaffolds to these reference assemblies with BLASTN [15] and used the best match in terms of the lowest E-value for each scaffold of the AMD data set as an estimate of its *correct taxonomic affiliation*.

**Figure 1:**
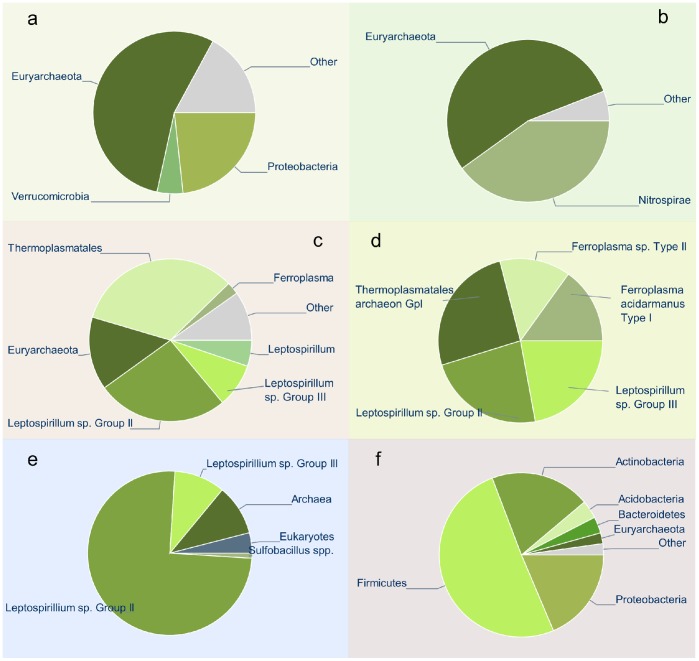
Taxonomic assignments of the acid mine drainage metagenome scaffolds. Each slice represents number of bases assigned. (a) the PhyloPythiaS generic model at the phylum level, (b) the PhyloPythiaS sample-specific model at the phylum level, (c) the PhyloPythiaS sample-specific model at various ranks, (d) taxonomic reference composition, obtained by alignment of the scaffolds with draft genome assemblies, (e) quantitative cell counts from a FISH study, reproduced from Tyson *et al.* (2004) [13] and (f) NBC with N-mer length 15 and Bacteria/Archaea genomes at the phylum level. The “Other” slice represents sequences that were unassigned or assigned at a higher level. Assignments were mapped to phylum level in plots a, b and f for ease of visualization.

We compared the PhyloPythiaS generic and sample-specific model assignments with predictions from the NBC web server (http://nbc.ece.drexel.edu/), MEGAN and the best BLASTN hit approach of MG-RAST (see Text S1). As MG-RAST and WebCARMA incorporate AMD sequences as reference data, a comparative evaluation by direct submission to these servers would not have ensured strict separation of the reference data and test data. Taxonomic scaffold assignments with PhyloPythiaS and the other tested methods were evaluated based on draft genome assemblies for the five strains and the Fluorescent In-Situ Hybridization cell counts published in the original AMD study (Figure 1d, e).

The PhyloPythiaS generic model returned the assignments in less than 5 minutes.Most scaffolds were assigned to high taxonomic ranks (taxonomic assignments are shown in Figure 1a, base-pair accuracy is given in Table 1; see Figures S1, S6 and Table S1). As no reference data were available in model construction for the sample populations, this was expected. Euryarchaeota were identified, but many scaffolds were assigned to phyla Proteobacteria and Verrucomicrobia, instead of to Nitrospirae. The generic model assignments were similar to those of BLASTN in terms of population abundance (Figure S3). In contrast, the NBC web server overestimated the abundance of Firmicutes and underestimated that of Euryarchaeota (Figure 1f, Figures S4 and S5).

**Table 1:** Percentage of bases correctly assigned to modeled taxa by different methods for the AMD metagenome scaffolds.

Rank	PhyloPythiaS sample-specific	PhyloPythiaS generic	BLASTN	MEGAN	NBC
**Genus**	41.353	0.000	0.000	0.000	0.000
**Family**	41.353	0.000	1.685	0.000	0.000
**Order**	74.706	38.189	45.536	42.210	1.742
**Class**	74.706	38.189	45.536	42.210	1.742
**Phylum**	89.540	47.821	47.011	42.673	1.798
**Domain**	92.673	88.978	86.042	70.194	44.805

The reference taxonomic affiliations were obtained by aligning the test scaffolds with the draft genomes. For PhyloPythiaS (both generic and sample-specific), the drop in accuracy is mostly due to unassigned sequences at a particular rank, while other methods produced more false assignments. Thermoplasmatales archaeon Gpl (comprising 21.8% of the total bases) has no defined parental clade at the genus and family ranks, contributing to the observed lower accuracy values for these ranks. Additional measures are shown in Figure S6.

For assignment using a sample-specific model, we randomly selected ∼100 kb of continuous sequences from the five populations as sample-specific training sequences. Specifically, the five strains and corresponding amounts of sample-specific data used were 70 kb for *Leptospirillum sp.* Group III, 100 kb for *Ferroplasma acidarmanus* Type I, 100 kb for *Leptospirillum sp. Group II '5-way CG'*, 100 kb for *Ferroplasma sp. Type II* and 70 kb for *Thermoplasmatales archaeon Gpl* (G-plasma). Construction of the sample-specific model took slightly less than 7 hours. The Newick tree and sample-specific data used to train the model are available on the web server as exemplary data. Assignments with this model (Figure 1b, c and Figure S2) corroborate well with the taxonomic makeup of this dataset. Both the generic and sample-specific models of PhyloPythiaS produced assignments that were taxonomically consistent and closer to the draft assemblies than those of the BLASTN approach, MEGAN and the NBC server (Table 2, Figure S6). Low scaffold consistency for the *Leptospirillum sp. Group II '5-way CG'* population (0.76) accompanied by low taxonomic distance between correct and predicted taxonomic affiliations (1.73) suggest that there was a certain degree of ‘back-and-forth’ in assignments between the Leptospirillum clades. In contrast, assignments for the Ferroplasma populations showed high scaffold consistency (>0.95) and higher taxonomic distance between correct and predicted affiliation (>3.7), suggesting that assignments were made to higher ranks (Table S1).

**Table 2:** Taxonomic distance analysis for AMD metagenome scaffolds assignment to draft genome assemblies generated for five strains of three different genera in the AMD metagenome project.

Method	Measure	Genus			Taxonomic Distance	
		L (543)	T (404)	F (236)	Micro average	Macro average
**PPS SS**	Assigned	528	404	236	–	–
	Const_n_scaff	0.92	0.83	0.97	0.89	0.91
	Const_n_bp	0.97	0.89	0.99	0.95	0.95
	Tax dist	0.96	1.79	2.22	1.48	1.65
**PPS G**	Assigned	540	403	236	–	–
	Const_n_scaff	0.36	0.81	0.95	0.63	0.71
	Const_n_bp	0.24	0.86	0.98	0.62	0.70
	Tax dist	6.90	1.96	2.53	4.32	3.80
**BLASTN**	Assigned	542	403	236	–	–
	Const_n_scaff	0.13	0.13	0.07	0.12	0.11
	Const_n_bp	0.06	0.08	0.02	0.05	0.05
	Tax dist	9.36	3.78	4.95	6.56	6.03
**MEGAN**	Assigned	337	272	194	–	–
	Const_n_scaff	0.60	0.14	0.12	0.22	0.28
	Const_n_bp	0.58	0.14	0.11	0.30	0.28
	Tax dist	5.77	2.12	3.93	2.78	3.94
**NBC**	Assigned	539	403	235	–	–
	Const_n_scaff	0.00	0.00	0.00	0.00	0.00
	Const_n_bp	0.00	0.00	0.00	0.00	0.00
	Tax dist	10.10	9.44	11.79	10.16	10.45

The genera are Leptospirillum (L), Thermoplasmatales (T), and Ferroplasma (F). The evaluated methods are the PhyloPythiaS sample-specific model (PPS SS), the PhyloPythiaS generic model (PPS G), BLASTN, MEGAN and the Naïve Bayesian classifier method (NBC). The assignments provided by each method were mapped to the genus or corresponding clade at a higher taxonomic rank for this analysis. The numbers in brackets after the population name show the number of scaffolds originating from each genus. The rows show the number of assigned scaffolds (Assigned), the fraction of scaffolds assigned to either the correct clade itself or a parental clade thereof (Const_n_scaff), the fraction of base-pairs in the same lineage as the correct taxon (Const_n_bp) and the average taxonomic distance of assignments with respect to genus level clades of the draft reference genomes (Tax Dist). See ‘Results’ for the definitions of consistency and taxonomic distance. Micro average shows average value over all test scaffolds and macro average shows average over the three genera.

### Analysis of a Metagenome Sample from Cow Rumen

**Figure 2:**
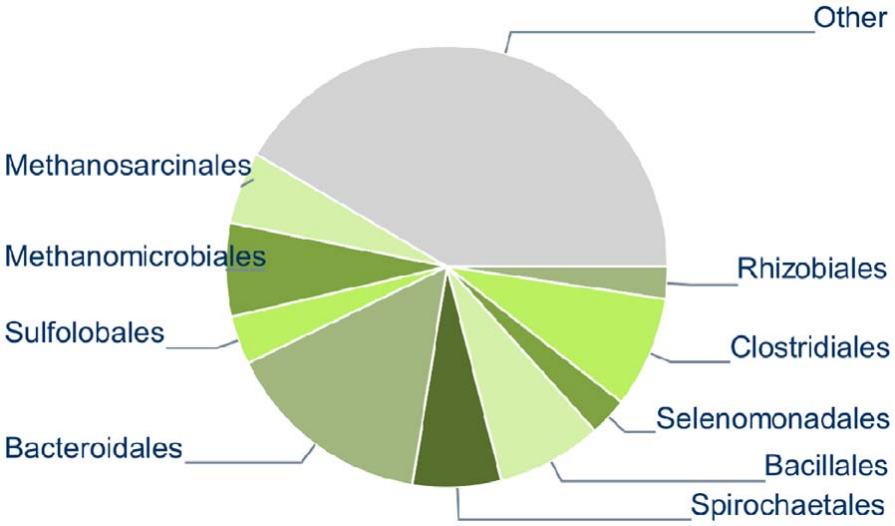
Taxonomic assignments of the cow rumen metagenome scaffolds with the PhyloPythiaS generic model. This data-set contained 26,042scaffolds in total. The assignments are shown at the order level. Each slice represents number of bases assigned. The “Other” slice represents sequences that were unassigned or assigned at a higher level.

We furthermore performed taxonomic assignments for 26,042 metagenomic scaffolds (568 Mbp) of a microbial community adherent to switchgrass incubated in a bovine rumen [14] with a twofold objective: First, to demonstrate usage of the server on a large dataset and, second, to verify usability of the method for sequences generated by Illumina sequencing technology. The data was downloaded from the DOE Joint Genome Institute website (ftp://ftp.jgi-psf.org/pub/rnd2/Cow_Rumen/). The majority of the scaffolds were found to have no similarity to sequenced genomes in the original study, suggesting uncharacterized microbes as their origin. We submitted the scaffolds to the web server in the generic mode as a multiplex sample and visualized the combined predictions. The majority of the scaffolds were assigned to the orders Bacteroidales, Clostridiales, Bacillales, Spirochaetales, Methanomicrobiales, Methanosarcinales, Sulfolobales, Selenomonadales and Rhizobiales (Figure 2).

Fifteen near-complete ‘genome bins’ of abundant populations from four orders were identified in the original study from the cow rumen sample, based on analysis of tetranucleotide frequency and assembly information [14]. We used these genome bins, comprising 466 scaffolds overall, as the *correct taxonomic affiliation* for comparison with the taxonomic assignments of PhyloPythiaS. The partial genome bins published in the original article are not guaranteed to be entirely correct, but provide a qualitative reference point, as they were generated based on multiple sources of information and verified by human in-depth inspection. We measured the assignment consistency as the number of base-pairs of these scaffolds consistently assigned by the PhyloPythiaS generic model to the order-level clades of the respective genome bins. Taxonomic distances of the predictions were calculated relative to the reported orders for the genome bins (Table 3). Overall, the generic model made consistent assignments for the majority of scaffolds. In particular, this was the case for genome bins of order-level clades with substantial numbers of reference genomes available, while assignment consistency was lower for clades covered by fewer reference genomes. Seven of the 15 bins were more than 90% consistent, four of them even to 100%. Five bins showed low consistency. In particular, we observed that the Clostridiales and Myxococcales genome bins were less consistent than bins of the other three orders. For Myxococcales this is likely because fewer sequenced genomes were available for training of the generic model (given the number of species with sequenced genomes for all five clades). For the Clostridiales, this might be due to genomic differences of the species represented by the genome bins to the sequenced Clostridiales genomes used as reference (mean GC content of 50% versus a mean GC content of 36%). However, regardless of the exact nature of the assigned taxonomic affiliation, scaffolds of a particular bin tended to be homogeneously assigned to the same clade by the generic model, varying from 44% to 100% of the scaffolds for the different bins. The predictive accuracy of the overall assignment can likely be further improved by construction of a sample-specific model, as we showed for the AMD sample.

**Table 3:** Taxonomic distance and consistency analysis of the 15 genome bins from the cow rumen metagenome consisting of 466 scaffolds in total.

Genome bin	Correct order	#Scaff	PhyloPythiaS generic model prediction		
			Tax Dist	Const_n_scaff	Const_n_bp
**AC2a**	Bacteroidales	20.000	0.000	1.000	1.000
**AJ**	Bacteroidales	22.000	0.000	1.000	1.000
**AMa**	Spirochaetales	19.000	0.000	1.000	1.000
**AQ**	Bacteroidales	24.000	0.000	1.000	1.000
**AH**	Bacteroidales	26.000	0.231	0.962	0.990
**ATa**	Clostridiales	32.000	0.625	0.906	0.967
**AGa**	Bacteroidales	35.000	0.743	0.886	0.938
**BOa**	Clostridiales	42.000	1.738	0.690	0.776
**AFa**	Spirochaetales	28.000	1.893	0.714	0.759
**APb**	Clostridiales	55.000	3.636	0.382	0.454
**AS1a**	Clostridiales	53.000	5.245	0.189	0.114
**AIa**	Clostridiales	22.000	6.682	0.182	0.086
**ADa**	Myxococcales	20.000	3.100	0.250	0.076
**AN**	Clostridiales	27.000	3.704	0.074	0.046
**AWa**	Clostridiales	41.000	7.073	0.000	0.000
**Macro average**	–	31.067	2.311	0.616	0.614
**Micro average**	–	–	2.693	0.560	0.613

The first three columns describe the dataset while the last three columns summarize the predictions of the PhyloPythiaS generic model. The last three columns show the average taxonomic distances between the predicted order and the correct order (Tax Dist), the consistency calculated based on the fraction of assigned scaffolds (Const_n_scaff) and the consistency calculated based on the fraction of assigned base-pairs (Const_n_bp). See ‘Results’ for the definitions of taxonomic distance and consistency. The micro average is the average value over all scaffolds and the macro average represents the average over the genome bins.

## Discussion

We provide a web server for taxonomic assignment of metagenome sequences with PhyloPythiaS. Software updates and custom-made models will be easily accessible to the community through the web server. Our server is unique in that it provides, in addition to generic models, the ability to build and use sample-specific models. The sample-specific mode allows additional sequences to be incorporated as a reference and relevant clades to be defined for a given community, e.g. based on accompanying 16S rRNA sample surveys. By taxonomic assignment of the AMD metagenome sample, we have shown how creation of such a sample-specific model allowed us to increase the coverage, resolution and accuracy of taxonomic assignments, with only a small amount (∼100 kb) of reference data being used. Due to computational limitations, no cross-validation for estimation of the hyperparameters is provided for sample-specific model construction, but our experiments show that default parameters produce accurate assignments on both simulated and real metagenome samples [8]. Furthermore, the assignments can be visualized and downloaded through an easy-to-use interactive interface. For the AMD metagenome, we found BLASTN (the strategy implemented by the MG-RAST server) to perform similarly to the generic model (both had an accuracy of >86% at the domain level), and the sample-specific model to show considerably improved assignment accuracy, in particular for lower taxonomic ranks. The NBC server mis-assigned a considerable fraction of the sequences and had an accuracy of ∼45% at domain level. MEGAN performed well on this data in terms of specificity, but showed lower sensitivity. To demonstrate use of the server and generic model for exploratory analysis of a large metagenome sample generated with the Illumina sequencing technology, we assigned scaffolds from the cow rumen metagenome in the generic mode. This showed high assignment consistency for the majority of the genome bins in comparison to a manual refined reference binning of the original study.

**Figure 3:**
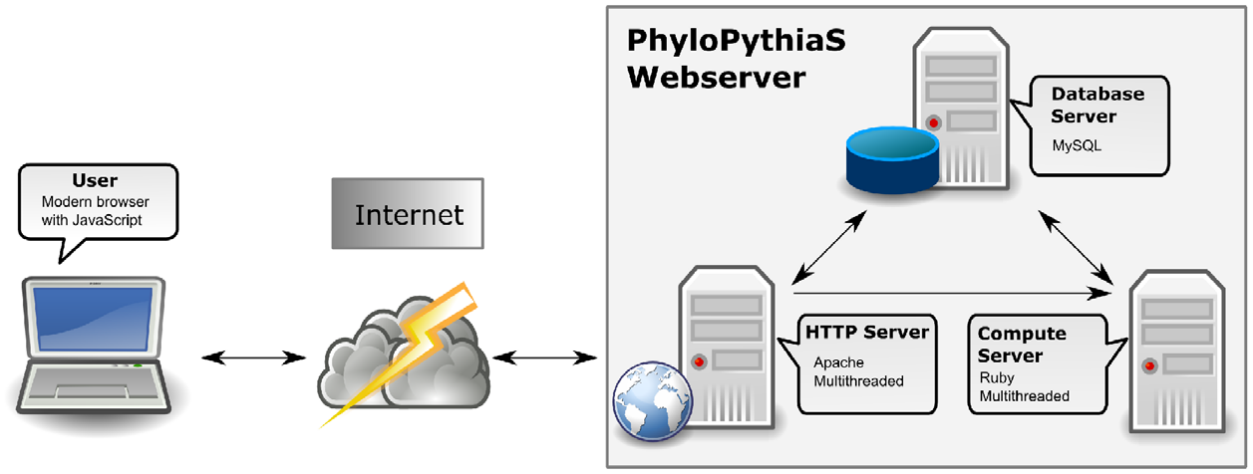
Schematic representation of the PhyloPythiaS web server implementation. Arrows represent the direction of communication.

With many high-throughput sequencing technologies being developed [16], it is important to assess how taxonomic assignment methods cope with the different technology-specific errors and read lengths. The technologies produce reads of different lengths and qualities, potentially affecting the performance of taxonomic assignment methods. We have previously shown [8] that PhyloPythiaS works well with assembled contigs from Sanger [17] and Roche/454 [18] sequencing technologies using metagenome samples from the Tammar wallaby gut [19] and from the guts of obese human twins [20], respectively. In the current study, we analyzed two datasets, one sequenced with Sanger and another with Illumina sequencing technology [21]. We found that regardless of the technology used, both of these datasets were characterized consistently. We expect the web server models to work equally well with assembled sequence data from other technologies with similar sequencing error rates, such as the SOLiD (Applied Biosystems) platform [22]. It should be noted that the performance of PhyloPythiaS on sequence fragments with high error rates is still unexplored. Furthermore, we advise that short reads should be assembled into longer contigs before submitting them to the PhyloPythiaS web server (see [23] for assembler comparisons). Although the server produces assignments for short sequences (<1000 bp), like with other methods, these assignments are less accurate than those for longer sequences and often to higher ranking taxa only. For scientists without access to large computing resources or familiarity with Unix/Linux, our server provides a novel, easily accessible resource for taxonomic assignment of metagenome sequence fragments.

## Materials and Methods

### PhyloPythiaS

The PhyloPythiaS model, referred to in short as the model hereafter, consists of an ensemble of structural support vector machines (SSVM) [24]. Each SSVM is induced using a sequence-composition derived input space and a taxonomy-based output space. By default, the model comprises six SSVMs, each induced on an input space that has been derived from *training fragments* of different length –1 kb, 3 kb, 5 kb, 10 kb, 15 kb and 50 kb. The input space is a combination of counts of substrings of length 4, 5 and 6 (k-mers), normalized based on the fragment length. The output space for each SSVM, defined by the taxonomy, is the same. At prediction time, a *test fragment* is classified using an ensemble of at most three SSVMs; built with fragments of the same length as the *test fragment* or longer.

PhyloPythiaS has two modes – generic and sample-specific. The generic mode uses a model trained with publicly available prokaryotic genomes and the taxonomy available at NCBI (http://www.ncbi.nlm.nih.gov/). Bacterial and archaeal taxa of seven major taxonomic ranks (species, genus, family, order, class, phylum and domain), with sequenced genomes from at least three genomes being available, were included in the reference taxonomy. The genomes were mapped to the lowest corresponding taxa of the model taxonomy and equal amounts of non-overlapping sequence fragments were selected for each taxon to create a training data set for each SSVM.

Lack of appropriate reference data can cause taxonomic assignments to be either of low resolution (i.e. assignments to high ranking taxa) or inaccurate. There are two reasons why the appropriate reference data might be lacking. First, the vast majority of microbial diversity has not been cultured and sequenced [25], and therefore metagenome samples often represent novel species for which no sequences of closely related organisms are available in public databases. Second, although genomic signature is informative for species and higher-level taxonomic clades [9], [26], it is also known that sequence characteristics are dependent upon environmental factors [27]. In this case, the genomic signature of the organisms in the metagenome sample can deviate from the genomic signature of the evolutionarily close organisms available in public databases. A sample-specific model (i.e. a model that includes training data from the metagenome sample itself in addition to public data) is better suited in such scenarios. By including sample-specific sequences and taxonomy in the training of SSVM, the dataset shift problem can be reduced [28]. Suitable sample-specific training sequences can be obtained from the metagenome sample itself, based on sequence homology to 16S rRNA or other phylogenetic marker genes, or by targeted sequencing of fosmids with such marker genes [29]. Trained with appropriate reference data, PhyloPythiaS allows the accurate assignment of sequence fragments with lengths of more than 1 kb, and is particularly well suited for the analysis of assembled sequence datasets. For shorter fragments, there is a loss in sensitivity, particularly at lower taxonomic ranks, which is a trend observed for all taxonomic assignment methods [8].

### Design and Implementation

As previously described, the web server can be used in two different modes – generic or sample-specific. The generic mode accepts sequences as a multi-FASTA file of up to 100 Mb in size and performs taxonomic assignments using a generic model. The generic model is constructed from prokaryotic genome sequences available at NCBI and models sufficiently covered clades from domain to species level (see Introduction). The sample-specific mode allows the user to specify the clades for a model and upload representative sequences for construction of a user-defined model. In this mode, the user has to provide three files: (1) a tree file: a plain text file with NCBI identifiers for the clades to be modeled or a rooted Newick tree with non-negative integer node names; (2) a sample-specific fasta file: a multi-FASTA file with sample-specific sequences, where each sequence header must contain a valid node identifier X as “label:X”; and (3) a prediction fasta file: a multi-FASTA file with the sequences for which taxonomic assignments are to be made. The sample-specific data provided by the user is pooled with the reference data used for generic model to build a model with default parameters as described in [8]. This model is then used for taxonomic assignment of the test sequences provided in the prediction fasta file.

The generic and sample-specific models produce output in the same format. The output page shows an assignments table with a maximum of 100 entries, as well as a pie chart and the model taxonomy. The pie chart shows the abundance of the taxa and can be interactively changed to visualize different taxonomic ranks and to display either the number of sequences or number of bases. The taxonomy shows the modeled tree along with the assignment information for each node. The taxonomy can be interactively changed to display either the taxonomic identifiers or the NCBI scientific names. This allows the user to easily visualize the distribution of the assignments over the taxonomy. Every node in the tree contains additional information, such as the number of sequences/bases assigned to the node or its subtree. Additionally, a link is provided to obtain the sequences assigned to each node. The assignments can be downloaded, possibly with additional data, or received via email. If the server was invoked in the sample-specific mode then additional assignments on separate data can be obtained using the same model.

Metagenome samples can be larger than the upload limitations of the web server. For this reason, the ability to visualize and download combined assignments from multiple submissions for classification with the same model is provided. One uploads a large sample in the form of multiple non-overlapping FASTA files, each as a different process, and retains the corresponding process identifiers. Once all the processes are finished, the process identifiers can then be provided to the ‘multiplex-sample’ utility, which combines the predictions from all processes and generates visualizations and download files.

The web server consists of multiple components (Figure 3). The web interface is implemented in PHP and JavaScript, and runs on an Apache server. The visualization and help routines are implemented in JavaScript using the Dojo toolkit (http://dojotoolkit.org/). The computational routines for the backend are written in the Ruby scripting language (http://www.ruby-lang.org/) embedded inside an XMLRPC server. These routines pre-process every job to create the necessary files and then invoke binaries compiled from C code (for k-mer feature generation and SSVM). A relational database based on MySQL is used to store the uploaded data, results and configuration. The jobs are processed in the same order they enter the database. The jobs and any associated data are deleted 30 days after their finishing time. The user does not need to register for using the server, and job identification and result retrieval is done using a unique identifier assigned to every job at submission time. By default, one processor each is reserved for the generic and the sample specific mode. This can be changed by administrators in case of large number of pending jobs and depending upon availability of resources.

### Availability

The PhyloPythiaS web server is freely available for non-commercial use at http://binning.bioinf.mpi-inf.mpg.de/.

## Supporting Information

Figure S1 **Assignments for the AMD metagenome scaffolds at different taxonomic ranks by the PhyloPythiaS generic model.** This model does not assign sequences to any of the genus level clades. This is expected behavior as none of the genera (Leptospirillum and Ferroplasma) were present in the generic model. The existence of Deltaproteobacteria (in Actual and Proteobacteria in Phylum) has been previously reported (reference [1] in Text S1) and is due to the provisional assignment of Leptospirillium to delta subdivision (reference [2] in Text S1).
(PDF)

Figure S2 **Assignments for the AMD metagenome scaffolds at different taxonomic ranks by PhyloPythiaS sample-specific model.** Sample-specific data (approximately 100 kb from each of the five strains) from the two genera (Leptospirillum and Ferroplasma) was used.
(PDF)

Figure S3 **Assignments for the AMD metagenome scaffolds at different taxonomic ranks by best BLASTN hit analysis.** E-value cut-off of 0.1 was used. The blast database used same genomes used for creating PhyloPythiaS generic model, i.e. all 1076 complete genomes available from NCBI as of April 2010.
(PDF)

Figure S4 **Assignments for the AMD metagenome scaffolds at different taxonomic ranks by the NBC webserver.** Default N-mer length of 15 with Bacteria/Archaea genomes were used. The webserver was accessed at http://nbc.ece.drexel.edu/in April 2011.
(PDF)

Figure S5 **Assignments for the AMD metagenome scaffolds fragmented at 500 bp at different taxonomic ranks by the NBC webserver.** To check for the possible effect of test sequence length on the taxonomic assignment of the AMD metagenome using the NBC webserver, we created fragments of length 500 bp from the scaffolds and obtained their assignments. Default N-mer length of 15 and Bacteria/Archaea genomes were used. Bacteria were overestimated while underestimating the Archaea. The NBC webserver was accessed at http://nbc.ece.drexel.edu/in May 2011.
(PDF)

Figure S6 **Performance of different methods at six major taxonomic ranks on the AMD data-set.** All the methods except PhyloPythiaS in sample-specific mode and BLASTN made only incorrect assignments at genus and family levels. The performance measures are used as defined in Patil et al. (reference [8] in the main text). The methods compared are the PhyloPythiaS generic model (PPS G), PhyloPythiaS sample-specific model (PPS SS), BLAST best hit (BLASTN), MEGAN and naïve Bayesian classifier (NBC).
(PDF)

Table S1 **Taxonomic distance analysis for AMD metagenome scaffolds assignment to draft genome assemblies generated for five strains in the AMD metagenome project.** The most specific assignments provided by each method were used for this analysis. The correct scaffold assignments, i.e. Population, were obtained using five strains (three species) whole genome shotgun sequences obtained from NCBI. The methods are PhyloPythiaS sample-specific model (PPS SS), PhyloPythiaS generic model (PPS G), BLASTN, MEGAN and naïve Bayesian classifier (NBC). The populations are *Thermoplasmatales archaeon Gpl* (T), *Leptospirillum sp. Group III* (L1), *Leptospirillum sp. Group II '5-way CG'* (L2), *Ferroplasma acidarmanus* (F1) and *Ferroplasma sp. Type II* (F2). The numbers in brackets after population name show number of correct scaffolds. The rows signify number of assigned scaffolds (Assigned), the fraction of assignments in the same lineage as the correct taxon (Const_n_scaff), the fraction of base-pairs in the same lineage as the correct taxon (Const_n_bp) and average taxonomic distance of with respect to draft reference genomes (Tax Dist).
(PDF)

Text S1 **Supporting text.**
(PDF)
