# Supplementary figures and images for: The PhyloPythiaS Web Server for Taxonomic Assignment of Metagenome Sequences
Source: PLoS One. 2012 Jun 20;7(6):e38581. doi: 10.1371/journal.pone.0038581 (PMC3380018; doi:10.1371/journal.pone.0038581)

## Actual assignment across ranks

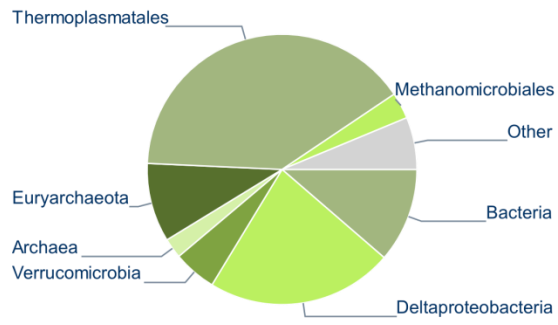

## Genus

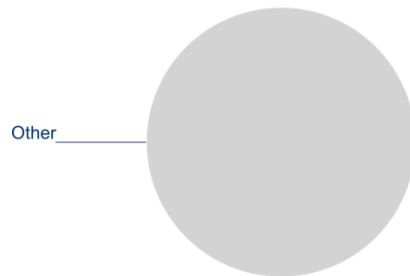

## Order

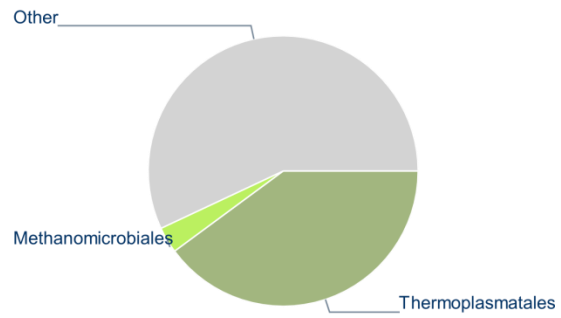

## Phylum

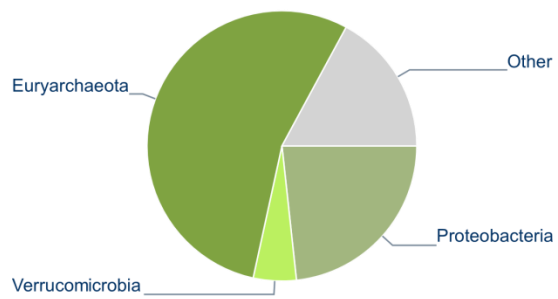

## Superkingdom

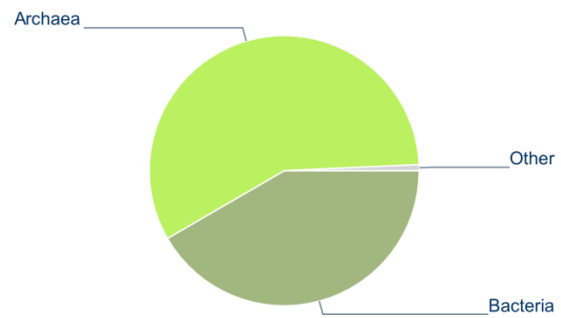

Supplement: Figure S1 — Assignments for the AMD metagenome scaffolds at different taxonomic ranks by the PhyloPythiaS generic model. This model does not assign sequences to any of the genus level clades. This is expected behavior as none of the genera (Leptospirillum and Ferroplasma) were present in the generic model. The existence of Deltaproteobacteria (in Actual and Proteobacteria in Phylum) has been previously reported (reference [1] in Text S1) and is due to the provisional assignment of Leptospirillium to delta subdivision (reference [2] in Text S1). (PDF) [file pone.0038581.s001.pdf]

## Actual assignment across ranks

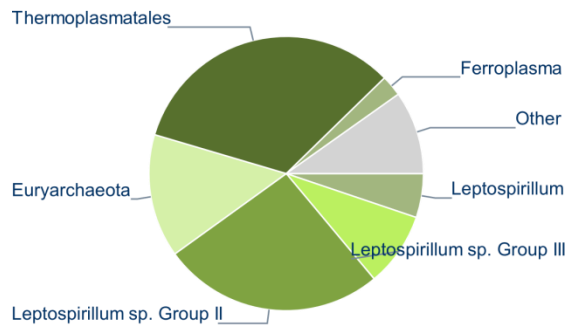

## Genus

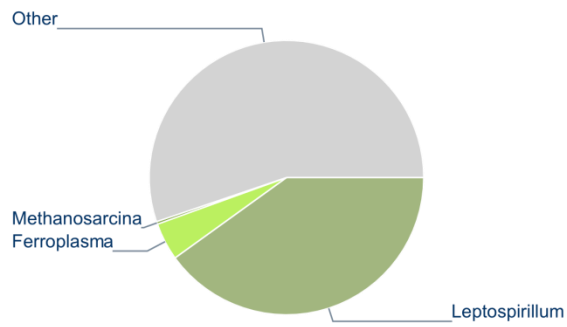

## Order

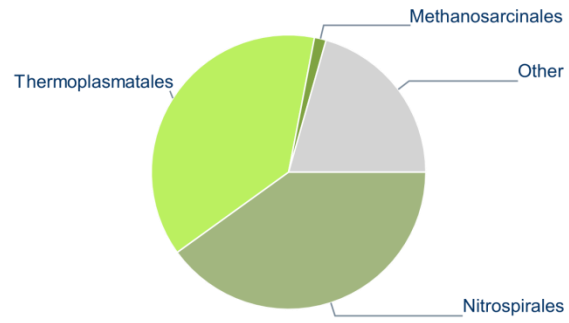

## Phylum

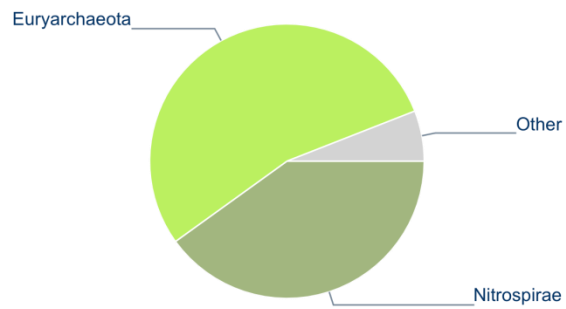

## Superkingdom

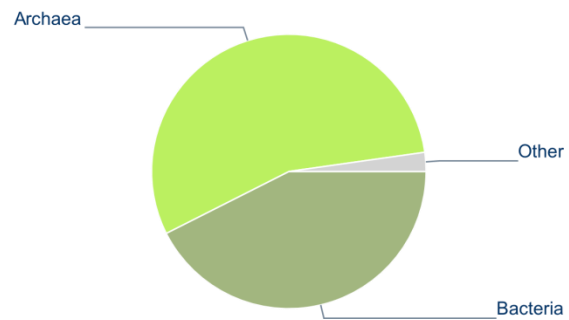

Supplement: Figure S2 — Assignments for the AMD metagenome scaffolds at different taxonomic ranks by PhyloPythiaS sample-specific model. Sample-specific data (approximately 100 kb from each of the five strains) from the two genera (Leptospirillum and Ferroplasma) was used. (PDF) [file pone.0038581.s002.pdf]

## Species/Strain

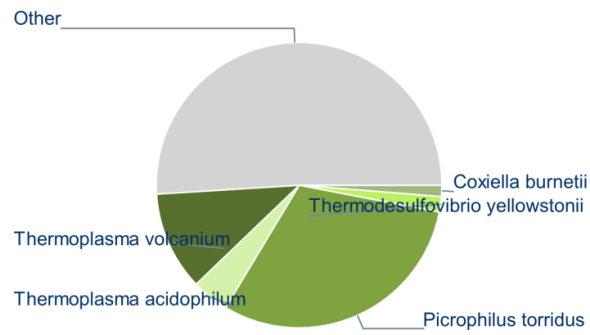

## Genus

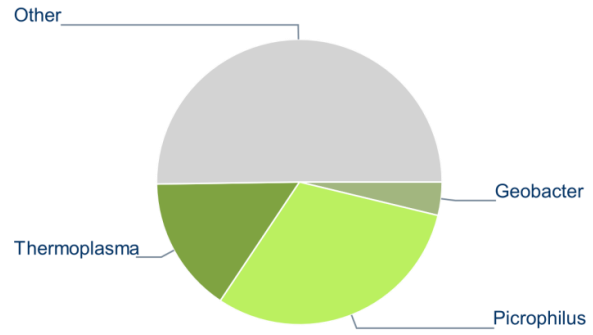

## Order

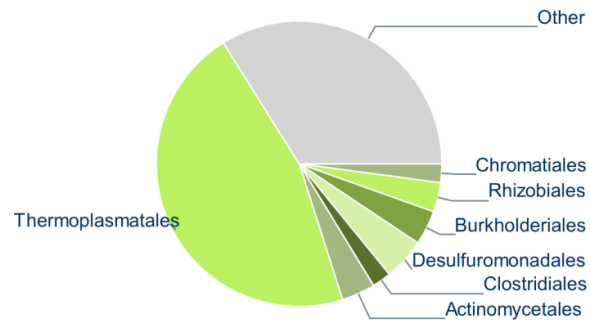

## Phylum

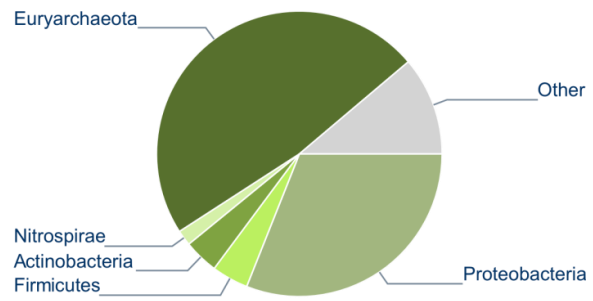

## Superkingdom

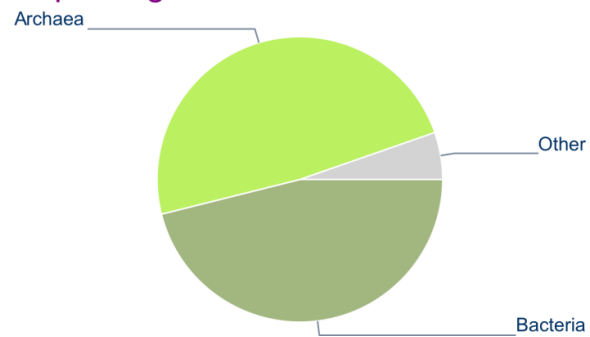

Supplement: Figure S3 — Assignments for the AMD metagenome scaffolds at different taxonomic ranks by best BLASTN hit analysis. E-value cut-off of 0.1 was used. The blast database used same genomes used for creating PhyloPythiaS generic model, i.e. all 1076 complete genomes available from NCBI as of April 2010. (PDF) [file pone.0038581.s003.pdf]

## Species

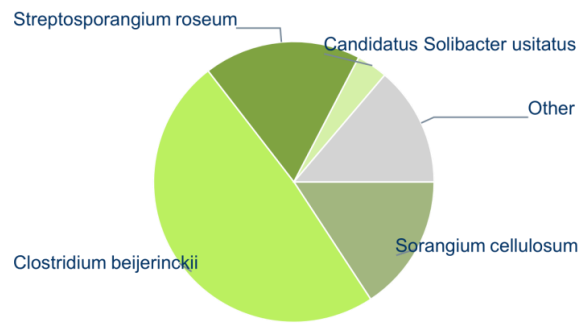

## Genus

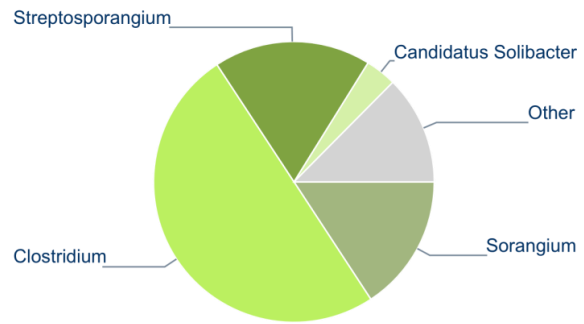

## Order

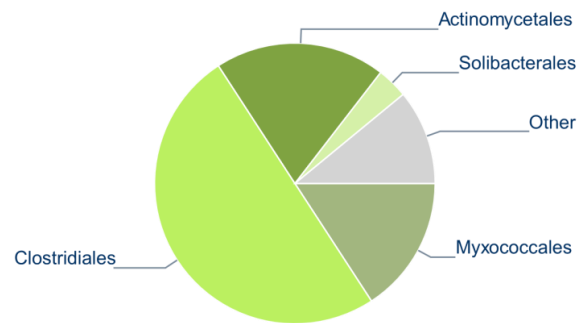

## Phylum

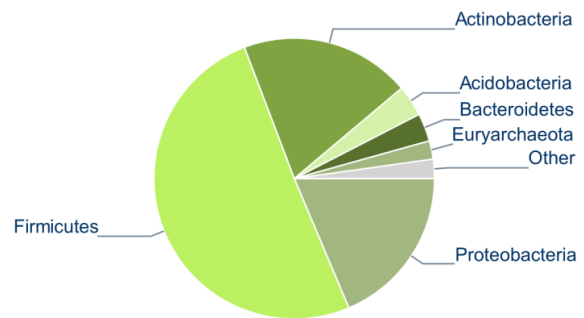

## Superkingdom

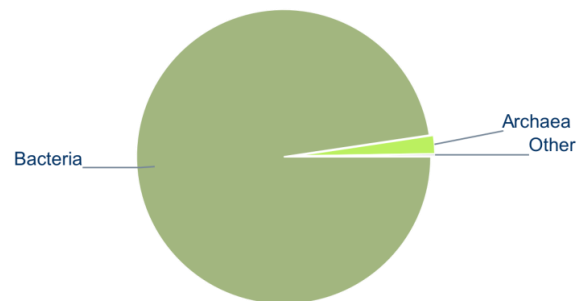

Supplement: Figure S4 — Assignments for the AMD metagenome scaffolds at different taxonomic ranks by the NBC webserver. Default N-mer length of 15 with Bacteria/Archaea genomes were used. The webserver was accessed at http://nbc.ece.drexel.edu/in April 2011. (PDF) [file pone.0038581.s004.pdf]

## Phylum

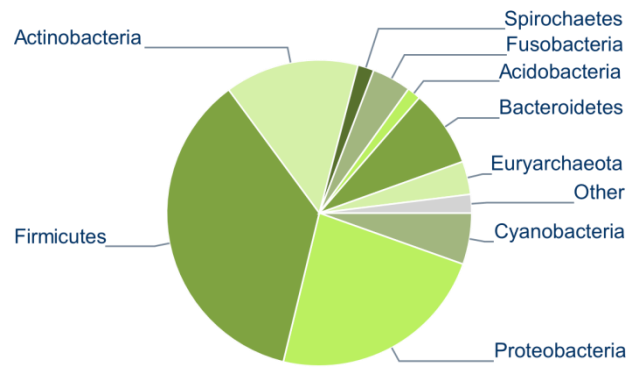

## Superkingdom

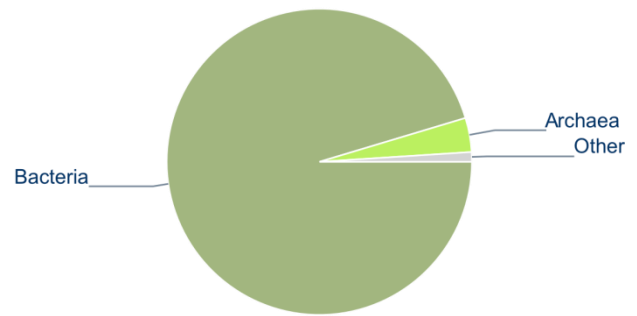

Supplement: Figure S5 — Assignments for the AMD metagenome scaffolds fragmented at 500 bp at different taxonomic ranks by the NBC webserver. To check for the possible effect of test sequence length on the taxonomic assignment of the AMD metagenome using the NBC webserver, we created fragments of length 500 bp from the scaffolds and obtained their assignments. Default N-mer length of 15 and Bacteria/Archaea genomes were used. Bacteria were overestimated while underestimating the Archaea. The NBC webserver was accessed at http://nbc.ece.drexel.edu/in May 2011. (PDF) [file pone.0038581.s005.pdf]

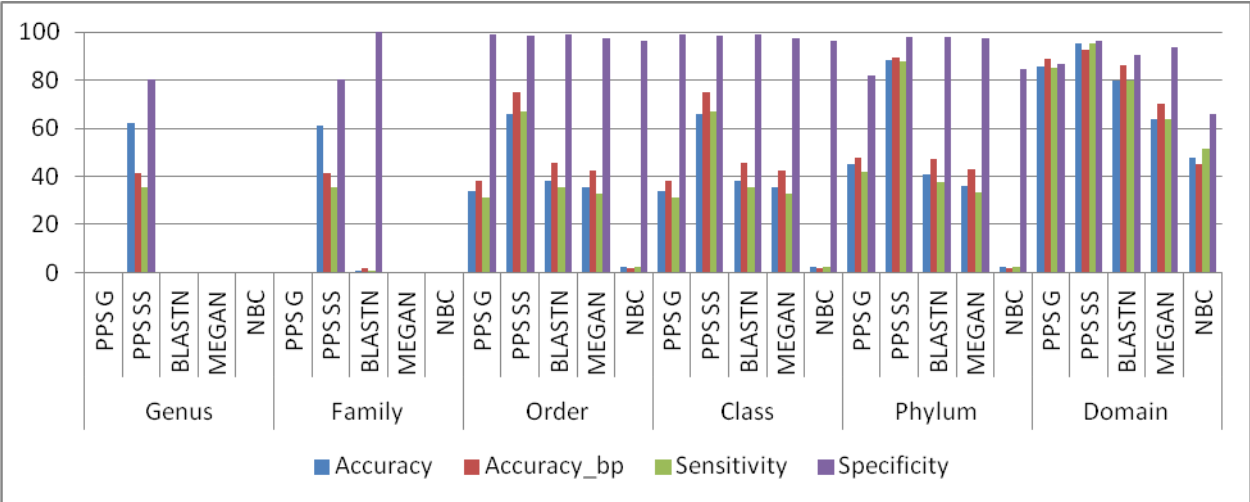

Supplement: Figure S6 — Performance of different methods at six major taxonomic ranks on the AMD data-set. All the methods except PhyloPythiaS in sample-specific mode and BLASTN made only incorrect assignments at genus and family levels. The performance measures are used as defined in Patil et al. (reference [8] in the main text). The methods compared are the PhyloPythiaS generic model (PPS G), PhyloPythiaS sample-specific model (PPS SS), BLAST best hit (BLASTN), MEGAN and naïve Bayesian classifier (NBC). (PDF) [file pone.0038581.s006.pdf]
