# Supplementary material for: The PhyloPythiaS Web Server for Taxonomic Assignment of Metagenome Sequences
Source: PLoS One. 2012 Jun 20;7(6):e38581. doi: 10.1371/journal.pone.0038581 (PMC3380018; doi:10.1371/journal.pone.0038581)
Supplement: Table S1 — Taxonomic distance analysis for AMD metagenome scaffolds assignment to draft genome assemblies generated for five strains in the AMD metagenome project. The most specific assignments provided by each method were used for this analysis. The correct scaffold assignments, i.e. Population, were obtained using five strains (three species) whole genome shotgun sequences obtained from NCBI. The methods are PhyloPythiaS sample-specific model (PPS SS), PhyloPythiaS generic model (PPS G), BLASTN, MEGAN and naïve Bayesian classifier (NBC). The populations are Thermoplasmatales archaeon Gpl (T), Leptospirillum sp. Group III (L1), Leptospirillum sp. Group II '5-way CG' (L2), Ferroplasma acidarmanus (F1) and Ferroplasma sp. Type II (F2). The numbers in brackets after population name show number of correct scaffolds. The rows signify number of assigned scaffolds (Assigned), the fraction of assignments in the same lineage as the correct taxon (Const_n_scaff), the fraction of base-pairs in the same lineage as the correct taxon (Const_n_bp) and average taxonomic distance of with respect to draft reference genomes (Tax Dist). (PDF) [file pone.0038581.s007.pdf]

Table S1

Taxonomic distance analysis for AMD metagenome scaffolds assignment to draft genome assemblies generated for five strains in the AMD metagenome project.

Method	Measure	Population					Taxonomic Distance	
		T (404)	L1 (417)	L2 (126)	F1 (172)	F2 (64)	Micro average	Macro average
PPS SS	Assigned	404	410	118	172	64	--	--
	Const_n_scaff	0.83	0.91	0.76	0.98	0.95	0.89	0.89
	Const_n_bp	0.89	0.94	0.95	0.99	0.99	0.94	0.95
	Tax dist	2.82	1.60	1.73	3.72	3.83	2.11	2.74
PPS G	Assigned	403	414	126	172	64	--	--
	Const_n_scaff	0.81	0.38	0.29	0.97	0.91	0.63	0.67
	Const_n_bp	0.86	0.38	0.11	0.99	0.98	0.62	0.66
	Tax dist	2.96	8.01	7.56	4.46	3.70	4.97	5.34
BLASTN	Assigned	403	416	126	172	64	--	--
	Const_n_scaff	0.13	0.16	0.05	0.07	0.08	0.12	0.10
	Const_n_bp	0.08	0.11	0.01	0.02	0.02	0.05	0.05
	Tax dist	5.65	11.18	11.45	7.97	6.64	7.90	8.58
MEGAN	Assigned	377	306	89	164	63	--	--
	Const_n_scaff	0.38	0.67	0.61	0.24	0.25	0.22	0.43
	Const_n_bp	0.33	0.65	0.57	0.19	0.12	0.37	0.37
	Tax dist	4.16	6.91	6.62	6.98	5.81	3.55	6.09
NBC	Assigned	403	413	126	172	63	--	--
	Const_n_scaff	0.00	0.00	0.00	0.00	0.00	0.00	0.00
	Const_n_bp	0.00	0.00	0.00	0.00	0.00	0.00	0.00
	Tax dist	11.35	10.97	10.65	14.85	13.63	12.40	12.29
