# Supplementary material for: The PhyloPythiaS Web Server for Taxonomic Assignment of Metagenome Sequences
Source: PLoS One. 2012 Jun 20;7(6):e38581. doi: 10.1371/journal.pone.0038581 (PMC3380018; doi:10.1371/journal.pone.0038581)
Supplement: Text S1 — Supporting text. (PDF) [file pone.0038581.s008.pdf]

Text S1

PhyloPythiaS generic model

The generic model is created using the complete genomes publicly available from NCBI. Six major taxonomic ranks, i.e. genus, family, order, class, phylum and domain, are used to create the taxonomy. The complete genomes are used to generate fragments of varying lengths, 1, 3, 5, 10 15 and 50 kb. Structured output support vector machine with a linear kernel is used to learn a classifier for each model. The combination of these six classifiers and an ensemble strategy is the PhyloPythiaS model. The ensemble uses lowest-node-maximum-votes strategy to combine the paths predicted by at-most 3 classifiers built using the closest or longer fragment length as that of the test sequence.

Complete genomes from NCBI

The analysis for the AMD data-set using PhyloPythiaS and BLASTN presented in this work was based upon the complete bacterial and archaeal genomes from NCBI GenBank (http://www.ncbi.nlm.nih.gov/genbank/) obtained in April 2010. This comprised of 1076 complete genomes in total. The currently available generic model was built using complete genomes downloaded from NCBI GenBank in May 2011 comprising 1332 genomes. The analysis of the cow rumen metagenome was conducted using the current generic model. This reference data will be periodically updated.

Analysis of results obtained from the NBC web server

We downloaded the assignments provided by the NBC webserver (http://nbc.ece.drexel.edu/, accessed in April 2011) and used the "summarized_results.txt" file to extract the sequence headers and species level assignments (columns 1 and 4). These assignments were used for subsequent analysis, for example generating pie charts and predictive performance calculations.

It might be that the NBC web server performs better on short sequence fragments rather than on long scaffolds. In order to check for this possibility, we created fragments of length 500 bp from the scaffolds and obtained their assignments. Default N-mer length of 15 and Bacteria/Archaea genomes were used. In this case, the NBC server was accessed in May 2011. The resulting assignments were mapped to phylum and domain level to facilitate visualization (Supplementary Figure 5). As with complete scaffolds (Supplementary Figure 4), bacterial clades were overestimated and archael clades were underestimated.

BLASTN best hit analysis

We created a BLAST [3] database, using "formatdb" command, with the 1076 complete genomes available from NCBI GenBank as of April 2010. The AMD scaffolds were queried, using "blastn", against this database with default parameters. The resulting blast report was parsed using Bioruby [4] and each scaffold was labeled with the taxonomic identifier of the genome with the best hit (lowest e-value). Hits with e-value less than 0.1 were discarded as being insignificant.

MEGAN analysis

MEGAN version 4.66.2 was downloaded from the website http://ab.inf.uni-tuebingen.de/software/megan/. The blast report was imported into MEGAN and taxonomic assignments were obtained using default parameters.

Pie chart generation

For PhyloPythiaS (generic and sample-specific) the webserver output page was used to obtain the pie charts. For other methods we used in house Ruby scripts (available upon request) to convert the assignments to a PhyloXML file that included the major taxonomic ranks (species, genus, family, order, class, phylum and superkingdom/domain) and additional information including, NCBI scientific names and number of sequences and bases assigned to each clade. The resulting phyloXML [5] file was then used to generate pie charts using the same JavaScript functions implemented in the PhyloPythiaS web server.

References

1. Bond PL, Smriga SP, Banfield JF (2000) Phylogeny of microorganisms populating a thick, subaerial, predominantly lithotrophic biofilm at an extreme acid mine drainage site. Applied and Environmental Microbiology 66: 3842-3849.
2. Bock E, Wagner M (2006) Oxidation of Inorganic Nitrogen Compounds as an Energy Source. In: Dworkin M, Falkow S, Rosenberg E, Schleifer K-H, Stackebrandt E et al., editors. The Prokaryotes: Springer New York. pp. 457-495.
3. Altschul SF, Gish W, Miller W, Myers EW, Lipman DJ (1990) Basic local alignment search tool. Journal of Molecular Biology 215: 403-410.
4. Goto N, Prins P, Nakao M, Bonnal R, Aerts J, et al. (2010) BioRuby: bioinformatics software for the Ruby programming language. Bioinformatics 26: 2617-2619.
5. Han MV, Zmasek CM (2009) phyloXML: XML for evolutionary biology and comparative genomics. Bmc Bioinformatics 10: 356.
